# Suicide in Psychiatric Inpatients— A Case–Control Study

**DOI:** 10.3389/fpsyt.2020.591460

**Published:** 2020-12-21

**Authors:** Eberhard A. Deisenhammer, Elisa-Marie Behrndt-Bauer, Georg Kemmler, Christian Haring, Carl Miller

**Affiliations:** ^1^Department of Psychiatry, Psychotherapy and Psychosomatics, Medical University of Innsbruck, Innsbruck, Austria; ^2^Department of Psychiatry and Psychotherapy, Center for Health Services Research in Medicine, Erlangen, Germany; ^3^Department of Psychiatry and Psychotherapy B, State Hospital Hall in Tirol, Hall in Tirol, Austria; ^4^Department of Psychiatry, County Hospital Kufstein, Kufstein, Austria

**Keywords:** suicide risk, in-patients, psychiatric hospital, hospitalization, admission

## Abstract

**Objective:** Psychiatric inpatients constitute a population at considerably increased risk for suicide. Identifying those at imminent risk is still a challenging task for hospital staff. This retrospective case–control study focused on clinical risk factors related to the course of the hospital stay.

**Method:** Inpatient suicide cases were identified by linking the Tyrol Suicide Register with the registers of three psychiatric hospitals in the state. Control subjects were patients who had also been hospitalized in the respective psychiatric unit but had not died by suicide. Matching variables included sex, age, hospital, diagnosis, and admission date. The study period comprised 7 years. Data were analyzed by the appropriate two-sample tests and by logistic regression.

**Results:** A total of 30 inpatient suicide cases and 54 control patients were included. A number of factors differentiated cases from controls; after correction for multiple testing, the following retained significance: history of aborted suicide, history of attempted suicide, history of any suicidal behavior/threats, suicidal ideation continuing during hospitalization, no development of prospective plans, no improvement of mood during the hospital stay, and leaving ward without giving notice. Logistic regression identified the latter three variables and history of attempted suicide as highly significant predictors of inpatient suicide.

**Conclusions:** Preventive measures during hospitalization include thorough assessment of suicidal features, an emphasis on the development of future perspectives, and a review of hospital regulations for patients who want to leave the ward.

## Introduction

Mental illness is associated with an increased risk for suicide (1–4). Psychiatric patients requiring admission to hospital treatment constitute a particularly vulnerable population given that they suffer the most severe subjective burden of disease. On the other side, psychiatric inpatients are placed in an environment which provides the most intensive professional therapeutic and caring setting and therefore should constitute the relatively safest one. In recent years, however, a debate as to whether hospitalization is actually protective against suicides or may even have risk-increasing effects on patient suicides has emerged (5, 6).

Inpatient suicide is defined as the suicide of a patient while hospitalized and also includes self-inflicted deaths occurring during a granted overnight or weekend leave if the continuation of the inpatient treatment was intended. A suicide occurring on the very same day of but after the patient's planned discharge is commonly (as far as the respective information is assessable) categorized as a post-discharge suicide.

Fortunately, inpatient suicides are relatively rare incidents. In a meta-analysis published a few years ago, the pooled estimate of suicide rates was 147 per 100,000 inpatient years (7). Related to admissions, rates range between 6 and 566 per 100,000 admissions (8). Recently, several studies suggested further decreasing rates, although some of these decreases may at least partially just be paralleling nationwide suicide rate declines (9–11). In contrast, Madsen et al. (12) found increases in inpatient suicide rates of 7.5% annually between 2009 and 2016 for Denmark. Still, the suicide of a hospitalized patient carries a significant emotional, sometimes even traumatic, load not only for the family, relatives, and friends but also for the hospital staff and fellow patients (13–15).

The prediction of a suicidal act, even during the period of inpatient treatment, where trained and experienced mental health staff is available day and night, still remains difficult (16). This is on one side due to the fluctuating nature of suicidal impulses and the reduced ability to communicate one's feelings inherent to suicidal individuals. On the other side, risk factors identified in studies oftentimes are distal ones, of limited specificity, or otherwise not utilizable for prevention (e.g., male gender, multiple hospitalizations, educational degree, and short duration of illness).

Case–control studies on inpatient suicides are scarce and had investigated varying aspects of suicide risk (17–20). In this study, we investigated risk factors for suicide during psychiatric hospitalization with a focus on variables assessed during the entire course of the hospital stay (i.e., clinical history, mode of and symptomatology at admission, and phase of hospitalization), i.e., predominantly proximal risk factors, comparing inpatient suicides with closely matched control patients.

## Methods

For this case–control study which was part of a larger project focusing on hospital suicides, data of all suicides which occurred in the Austrian state of Tyrol between 01-Feb-2004 and 31-Jan-2011 were obtained from the Tyrol Suicide Register (TSR). The study period thus comprised 7 years during which a total of 775 suicides were documented in the TSR. The TSR was linked with the hospital registers of the, at that time, three psychiatric units in Tyrol [the respective departments of a university hospital in Innsbruck (UHI), a state hospital in Hall in Tirol (SHH), and a county hospital in Kufstein (CHK)]. Through this procedure, 30 inpatient suicide cases (3.9% of the total number) were identified.

Controls were defined as psychiatric hospital inpatients during the same period who had not died by suicide. Based on the decision to focus on clinical factors related to the hospital stay, a close matching strategy was chosen. Matching variables included sex, age (±max. 9 years), hospital, diagnosis (according to ICD-10 chapters: F1, F2, F3, etc.), and admission date (±max. 1 week). For each case, the respective closest potential controls were chosen. It was aimed to identify two controls for each case. Due to the thorough matching strategy, however, it was not possible to attain the targeted 60 controls, but only 54.

Patient and admission-related data were extracted from available hospital records (e.g., admission record, assessment for restraint measures, and documentation during stay) and included demographic and clinical history variables, symptoms at admission, mode of admission, treatment variables, and symptoms in the last record. Data were extracted by one of the authors only (E-MB-B), a psychologist clinically trained to assess psychopathological alterations.

The study procedure was approved by the Ethics Committee of the Medical University of Innsbruck.

### Statistical Analysis

The two groups (inpatient suicides and controls) were compared with regard to the relevant demographic, clinical, and admission-related variables by means of the appropriate two-sample tests. The chi-square test was used for categorical variables, and Student's *t*-test or Mann–Whitney *U*-test for continuous variables depending on their distribution (approximately normal or non-normal, respectively). To quantify group differences, effect sizes and corresponding 95% confidence intervals (CI) were calculated, Cohen's *d* for metric variables and odds ratios (ORs) for dichotomous variables. Statistical testing was performed at a significance level of 0.05 and also at an adjusted α-level to correct for multiple testing by means of the Bonferroni method (α_adj._ = 0.05/number of tests performed). Both uncorrected and Bonferroni-corrected test results are reported.

To investigate the combined effects of the above variables, we performed a logistic regression analysis with group (inpatient suicide vs. control) as the dependent variable. To avoid problems with multiple testing, only those variables that had attained significance in the univariate analysis with Bonferroni correction were entered as independent variables. Significant predictors were determined by forward stepwise variable selection using the likelihood ratio test. As most of the ORs obtained in the analysis were undetermined due to almost complete separation [some combinations of predictors gave rise to estimated suicide risks of 0% or 100%, see (21)], we summarized our findings in a way that is different than usual. We formed a sum score, *S*, based on the four significant risk factors, by counting how many of these risk factors were present in the individual patient. Confidence intervals (CIs) for the suicide risk in dependence of *S* were determined by means of the adjusted Wald method.

## Results

Demographic and clinical data of inpatient suicides and controls are displayed in Table 1. The proportion of females was 60 and 57.4%, respectively. Two thirds of the suicides had occurred in the largest of the three units, the state hospital (SHH). The most frequent diagnosis in both groups was a mood disorder (ICD-10: F3; 70%). Jumping from a height accounted for 40% of the suicide methods applied, followed by hanging and jumping in front of a train (both 23.3%).

**Table 1:** Demographic and clinical data of suicide and control patients.

**Variable**	**In-patient suicides**	**Controls**	**Test statistic^a^**	***p*-value**
	**(*n* = 30)**	**(*n* = 54)**	**(χ^2^ or *t*)**	
	***n* (%) or ø ± SD**	***n* (%) or ø ± SD**		
Sex (males/females)	18 (60%)/12 (40%)	31 (57.4%)/23 (42.6%)	χ^2^ = 0.05	0.817
Age (years)	52.0 ± 14.8	50.4 ± 14.8	*t* = 0.46	0.645
**HOSPITAL**				
UH Innsbruck	6 (20%)	9 (16.7%)	χ^2^ = 0.16 df = 2	0.923
SH Hall	20 (66.7%)	38 (70.4%)		
CH Kufstein	4 (13.3%)	7 (13.0%)		
**MAIN DIAGNOSIS AT DISCHARGE (ICD-10 CHAPTERS)**				
F0	1 (3.3%)	2 (3.7%)	χ^2^ = 5.42 df = 5	0.367
F1	1 (3.3%)	3 (5.6%)		
F2	3 (10%)	3 (5.6%)		
F3 (F31/F32/F33)	21 (5/8/8) (70%)	38 (11/17/10) (70.4%)		
F4/F6	4 (13.4%)	8 (14.8%)		
**SUICIDE METHODS**				
Jumping from a height	12 (40%)			
Hanging	7 (23.3%)			
Jumping in front of a train	7 (23.3%)			
Drowning	3 (10%)			
Cutting	1 (3.3%)			

*n.s., not significant (p > 0.05)*.

a *Chi-square test (χ^2^) or t-test (t)*.

Nine suicides (30%) occurred inside the hospital itself and only three (10% of the total) in a closed ward. Ten suicides (33.3%) occurred within 1 week after admission, six of these within 3 days and two on the day after admission. Sixteen suicides (53.3%) occurred after the patients had left the ward without informing the staff.

The total inpatient suicide rate was 45.7 per 100,000 admissions. The rates differed considerably between the three units [UHI, 21.8; SHH, 62.8; CHK, 63.1; significant for UHI vs. SHH only (*p* = 0.018)].

Variables grouped according to phase of hospital stay (i.e., clinical history, admission, course of hospital stay, and day of discharge) are displayed in Tables 2, 3. In short, patients who died by suicide had significantly more often a history of aborted suicide attempts and of any suicidal behavior. They further reported more often suicidal ideation, depressive symptoms, and a current stressor at admission but showed less often aggressive behavior. Suicide patients were significantly more often referred from a nonpsychiatric unit and had less often made contact with the hospital by themselves. They were less often able to develop prospective plans for the time after discharge. In the respective last documentation, we found less often mood improvement and more often suicidal ideation. Only one of the control patients had left the ward without giving notice during the hospital stay while more than half of the inpatient suicide subjects had left the ward without notification at least once.

**Table 2:** Pre-admission clinical history, symptoms at admission and mode of admission.

**Variable**	**In-patient suicides**	**Controls**	**Effect size**	**95% confidence interval**	***p*-value^a^**
	**(*n* = 30)**	**(*n* = 54)**	**(Cohen's *d* or OR)**		
	***n* (%) or ø ± SD**	***n* (%) or ø ± SD**			
**PRE-ADMISSION**					
Number of previous admissions	3.9 ± 3.1	4.6 ± 6.6	*d* = −0.12	(−0.57 to 0.33)	n.s.
History of attempted suicide	20 (66.7%)	6 (11.1%)	OR = 16.0	(5.13–50)	<0.001^#^
Aborted suicide attempt	8 (26.7%)	1 (1.9%)	OR = 19.3	(2.27–163)	<0.001^#^
Any suicidal behavior/threats	25 (83.3%)	22 (40.7%)	OR = 7.27	(2.41–21.9)	<0.001^#^
**SYMPTOMATOLOGY AT ADMISSION**					
Current suicide attempt	3 (10%)	1 (1.9%)	OR = 5.89	(0.58–59.0)	n.s.
Suicidal ideation	12 (40%)	10 (18.5%)	OR = 2.33	(1.08–7.99)	0.032^*^
Anxiety	6 (20%)	16 (29.6%)	OR = 0.59	(0.20–1.73)	n.s.
Depressive symptoms	28 (93.3%)	41 (75.9%)	OR = 4.44	(1.01–21.2)	0.046^*^
Current stressor (partnership, work, death)	16 (53.3%)	16 (29.6%)	OR = 2.71	(1.08–6.85)	0.032*
Currently under the influence of alcohol	3 (10.3%)	8 (14.8%)	OR = 0.66	(0.16–2.70)	n.s.
Other substances	4 (13.3%)	4 (7.4%)	OR = 1.92	(0.44–8.32)	n.s.
Thought disorder (content)	7 (24.1%)	15 (27.8%)	OR = 0.83	(0.29–2.33)	n.s.
Cognitive impairment	20 (66.7%)	33 (61.1%)	OR = 1.27	(0.50–3.24)	n.s.
Aggression	1 (3.3%)	12 (22.2%)	OR = 0.12	(0.02–0.98)	0.022*
**WAY OF ADMISSION**					
Brought to the hospital by ambulance	9 (30%)	10 (18.5%)	OR = 1.89	(0.66–5.33)	n.s.
Police	2 (6.7%)	5 (9.3%)	OR = 0.70	(0.13–3.85)	n.s.
Involuntary admission (“Parere”)	1 (3.3%)	5 (9.3%)	OR = 0.34	(0.04–3.04)	n.s.
Referral by physician	20 (66.7%)	28 (51.9%)	OR = 1.86	(0.73–4.70)	n.s.
from another psychiatric unit	1 (3.3%)	0 (0%)	–^b^	–^b^	n.s.
from a not psychiatric unit	5 (16.7%)	1 (1.9%)	OR = 10.60	(1.12–95)	0.020*
Self-referral by patient	5 (16.7%)	24 (44.4%)	OR = 0.25	(0.08–0.75)	0.010*
Patient accompanied by another person	11 (40.7%)	19 (35.2%)	OR = 1.27	(0.49–3.27)	n.s.

*n.s., not significant (p > 0.05); OR, odds ratio; d, Cohen's effect size d*.

a *Mann-Whitney U-test (number of previous admissions) or Chi-square test (all other variables)*.

b *Odds ratio undetermined*.

* *Statistically significant at the 0.05 level. However, significance is not retained after correction for multiple testing*.

# *Statistical significance is retained after correction for multiple testing*.

**Table 3:** Factors during hospitalization and symptoms in last record.

**Variable**	**In-patient suicides**	**Controls**	**Effect size**	**95% confidence interval**	***p*-value**
	**(*n* = 30)**	**(*n* = 54)**	**(Cohen's *d* or OR)**		
	***n* (%) or ø ± SD**	***n* (%) or ø ± SD**			
**FACTORS DURING HOSPITALIZATION**					
Duration of hospitalization (days)	21.1 ± 20.3	23.1 ± 18.4	*d* = −0.10	(−0.56 to 0.36	n.s.
Days in closed unit	11.1 ± 9.4	11.9 ± 9.5	*d* = −0.08	(−0.54 to 0.38	n.s.
Initially closed unit	11 (36.7%)	13 (24.1%)	OR = 1.83	(0.69–4.82)	n.s.
Change between wards	9 (30%)	12 (22.2%)	OR = 1.50	(0.55–4.12)	n.s.
Refusal of therapeutic interventions	8 (26.7%)	12 (22.2%)	OR = 1.27	(0.45–3.57)	n.s.
Life event	4 (13.3%)	3 (5.6%)	OR = 2.63	(0.54–12.6)	n.s.
Prospective plans (job situation, housing, partnership)	7 (23.3%)	48 (96%)	OR = 0.01	(0.002–0.07)	<0.001^#^
**SYMPTOMS IN LAST RECORD**					
Improvement of mood	14 (51.9%)	51 (94.4%)	OR = 0.06	(0.01–0.25)	<0.001^#^
Suicidal ideation	9 (30%)	0 (0%)	–^b^	–^b^	<0.001^#^
Left ward without giving notice	16 (53.3%)	1 (1.9%)	OR = 60.6	(7.39–496)	<0.001^#^
Co-morbid diagnosis	16 (53.3%)	26 (48.1%)	OR = 1.23	(0.50–3.01)	n.s.

*n.s., not significant (p > 0.05); GP, general practitioner*.

a *Mann-Whitney U-test (durations) or Chi-square test (all other variables)*.

b *Odds ratio undetermined*.

# *Statistical significance is retained after correction for multiple testing*.

After correction for multiple testing, the following variables retained significance: history of aborted suicide, history of any suicidal behavior or threats, no prospective plans, no improvement of mood, suicidal ideation continued during hospitalization, and leaving ward without giving notice.

The combined effects of the above variables on the risk of inpatient suicide were investigated by logistic regression. Of the independent variables taken into account (see Statistical Analysis), the following four emerged as significant predictors of an increased suicide risk: history of attempted suicide, leaving ward without notice, no prospective plans (always χ^2^ > 14, *p* <0.001), and no improvement of mood at discharge (χ^2^ = 7.36, *p* = 0.007). Table 4 shows how the risk of suicide increases with the number of risk factors. Among the patients without any of these risk factors, there were no suicides. The corresponding CI indicates a rather small risk of suicide (95% CI: 0–7.6%). Patients with one risk factor had a higher suicide risk (estimate of 28.6%). In the subgroup with two or more risk factors, all patients belonged to the inpatient suicide group. The corresponding 95% CIs indicate that the suicide risk in this group was very high: 75–100% for patients with two and 79–100% for those with three to four risk factors.

**Table 4:** Risk of inpatient suicide in dependence of the number of risk factors.

**Risk score, *S* (number of risk factors present^a^)**	**Total^b^**	**Controls**	**In-patient suicides**	**Suicide risk (95% CI, adjusted Wald method)**
0	40 (100%)	40 (100.0%)	0 (0.0%	0.0% (0.0–7.6%)
1	14 (100%)	10 (71.4%)	4 (28.6%)	28.6% (11.3–55.0%)
2	10 (100%)	0 (0.0%)	10 (100.0%)	100.0% (75.1–100.0%)
3–4	13 (100%)	0 (0.0%)	13 (100.0%)	100.0% (79.7–100.0%)

a *Risk factors taken into account: History of attempted suicide, left ward without giving notice, no prospective plans, no improvement of mood*.

b *Seven patients had to be excluded due to not reported observations on an least one of the risk factors*.

## Discussion

The data presented here are part of a larger project investigating hospital-associated suicides in the state of Tyrol. A similar case–control procedure was used for a study in post-discharge suicides (i.e., suicides occurring within 12 weeks after discharge from a psychiatric hospital), which identified, among others, a history of suicidal behavior or threats, depression and thought disorder at admission, a change between wards during hospitalization, depression at discharge, and lack of a fixed appointment with a general practitioner for post-discharge care as suicide risk factors (22). In another study using data from this project, inpatient suicides were compared with post-discharge cases and suicides not recently hospitalized with the result of between-group differences in several variables including gender ratio and suicide method distribution (23).

In the present study in psychiatric inpatients focusing on clinical factors assessed during the entire hospitalization period, we found a number of significant differences between inpatient suicides and control subjects from which clinical recommendations for suicide prevention measures can be derived.

Of the factors retaining statistical significance after correction for multiple testing, a history of previous suicidal behavior (including aborted suicide attempts) or suicidal threats is one that has previously been reported in a number of studies. This risk factor is true not only for inpatient suicides (18, 24–27) but also for post-discharge suicides (22, 28, 29). To address this issue, including a thorough assessment of previous suicidal ideation and behavior into the admission interview remains an important measure for the identification of potentially suicidal patients (30). In addition, this information should be kept in mind throughout the entire therapeutic process during the hospital stay and even beyond.

Several factors relating to symptoms at admission and the mode of admission lost statistical significance when applying a Bonferroni correction for multiple testing, possibly due to limited statistical power. However, most of these still appear to be plausible puzzle pieces in the risk profile of suicidal inpatients. These include suicidal ideation, depressive symptoms, a current stressor (in relation to partnership, work, or loss of a significant other), and less aggressive features at admission as well as being transferred from a nonpsychiatric unit more often and having contacted the hospital by oneself less often. While suicidal and depressive symptoms assessed at admission have been reported in previous studies (19, 20, 27) and are well comprehensible, less aggression noted in the admission record appears to be less obvious. However, although aggression has been related to suicidal behavior (31, 32), this association is not unequivocal (33). From a psychodynamic point of view, the defense (or—in Adlerian terminology—safety) mechanism of turning inhibited aggression toward the self (34) may obscure aggressive impulses and thus result in a seemingly low-aggression appearance.

For patients who later died by suicide, treating physicians had less often stated in the records that they had generated prospective plans concerning their job situation, housing conditions, or partnership during the hospital stay. In recent years, research has emphasized the importance of such socioeconomic (in addition to clinical and psychological) factors for the occurrence of suicidal ideation and behavior (35–38). In the therapeutic setting in hospitals, it appears of utmost importance to include the interaction of depressive symptoms and other clinical features with the circumstances of the patient's life into treatment planning. Further, a focus should be laid on the development of perspectives to help the patient to free oneself from the suicidal entrapment.

The poorer course of inpatient suicide cases during the hospital stay compared with controls is also reflected by the psychopathological load described in the respective last patient records. Improvement of mood was less often documented and suicidal ideation was more often documented for patients who died by suicide shortly after.

We also found more often a record of a nonarranged leaving of the ward while hospitalized. Such a behavior during the hospital stay has been reported in previous studies to be associated with an increased suicide risk in inpatients (18, 39–41). As a clinical preventive measure, it might be worth considering to review the respective hospital regulations for patients who want to leave the ward, e.g., for a walk or for smoking. For example, an obligatory presentation to a nurse before leaving the ward may reveal short-term behavioral alterations and lead to at least some form of communication (which is probably the most effective suicide preventive measure in an acute situation). As a further recommendation, establishing standard operating procedures in case a patient absconds from the ward (e.g., having a list of all phone numbers of the patient and significant others at hand, informing the hospital security service, or prompting a search by police) can be derived from these findings.

More than two thirds of the suicides happened outside the hospital. This is in line with the results of previous research (17, 41, 42) and supports the view of a general protective effect of being under professional observation. The hospital setting also, and probably even more importantly, facilitates the possibility to get into contact and communication with other people. This is provided on a ward by trained hospital staff more or less at any time, when suicidal urges acutely emerge.

The inpatient suicide rate of 45.7 per 100,000 admissions found in this study is a considerable reduction of about two thirds compared to the rate of 132 reported in a study we had conducted about 20 years ago in two of the hospitals included here (43). In contrast, the general suicide rate of Tyrol during the past decades decreased to a much lesser extent, from 21.9/100.000 inhabitants in 1995 to 16.4 in 2019. There was also no significant change in the number of psychiatric hospital beds in the region. The inpatient suicide rate decrease, which was also found in other European countries (44, 45), may thus be an actual consequence of suicide preventive measures in psychiatric hospitals.

There were considerable and in part significant differences in inpatient suicide rates between the three hospitals (UHI, 21.8 per 100,000 admissions; SHH, 62.8; CHK, 63.1). Differences between categories of hospitals have so far been reported for post-discharge suicides only (22, 46). Potential explanations for the variability in inpatient suicide rates include different patient populations, differences in hospital safety measures, varying patient-to-physician/psychotherapist/nurse ratios, differing regulations concerning overnight and weekend leaves, and many more. It should be noted, however, that case numbers in two of the hospitals were low.

In general, the relatively low number of suicide cases is one of the limitations of this study. Further, clinical information was not assessed with standardized instruments or scales but was extracted retrospectively from routine records, thus, on the other side, reflecting real-world availability of clinical data. Related to this, the required information may not have been documented for all patients, thus potentially leading to some degree of underassessment. The main strength of this study is the thorough matching strategy making it possible to focus on factors associated with the hospital stay, the time immediately preceding suicide.

In conclusion, in this study on psychiatric inpatient suicides, we found a number of factors associated with the risk of suicide. Preventive measures that can be derived from these results include a thorough and periodical assessment of suicidal features during the entire phase of hospitalization (and beyond), an emphasis on the development of future perspectives in the therapeutic working with the patient, and a review of hospital regulations for patients who want to leave or have absconded from the ward.

## Data Availability Statement

The datasets presented in this article are not readily available because Personal data. Requests to access the datasets should be directed to Eberhard A. Deisenhammer, eberhard.deisenhammer@i-med.ac.at.

## Ethics Statement

The studies involving human participants were reviewed and approved by Ethics Committee of the Medical University of Innsbruck. Written informed consent for participation was not required for this study in accordance with the national legislation and the institutional requirements.

## Author Contributions

EAD: design of the study writing. E-MB-B: extracting of data. CH: extracting of data. CM: extracting of data. GK: statistics. All authors contributed to the article and approved the submitted version.

## Conflict of Interest

The authors declare that the research was conducted in the absence of any commercial or financial relationships that could be construed as a potential conflict of interest.
